# Laser Therapy for Vascular Malformations of the Oral Cavity: A Systematic Review

**DOI:** 10.3390/dj13090416

**Published:** 2025-09-09

**Authors:** Matteo Pellegrini, Martina Bosisio, Federica Pulicari, Carmen Darinca Todea, Francesco Spadari

**Affiliations:** 1 Department of Biomedical, Surgical and Dental Sciences, University of Milan, Via della Commenda 10, 20122 Milan, Italy; martina.bosisio1@studenti.unimi.it (M.B.); federica.pulicari@unimi.it (F.P.); francesco.spadari@unimi.it (F.S.); 2 Maxillofacial Surgery and Dental Unit, Fondazione IRCCS Cà Granda Ospedale Maggiore Policlinico, 20122 Milan, Italy; 3 Section of Dentistry, Department of Clinical, Surgical, Diagnostic and Pediatric Sciences, University of Pavia, 27100 Pavia, Italy; 4 Department of Oral Rehabilitation and Dental Emergencies, Faculty of Dentistry, “Victor Babes” University of Medicine and Pharmacy, Eftimie Murgu Square No. 2, 300041 Timisoara, Romania; todea.darinca@umft.ro; 5 Interdisciplinary Research Center for Dental Medical Research, Lasers and Innovative Technologies, Revolutiei 1989 Avenue No. 9, 300070 Timisoara, Romania

**Keywords:** CO_2_ laser, diode laser, Er,Cr:YSGG laser, laser therapy, Nd:YAG laser, oral vascular malformations

## Abstract

**Objectives**: to compare the clinical effectiveness, safety, and aesthetic outcomes of different laser systems used for the treatment of oral vascular malformations. **Materials and Methods**: This review followed JBI guidelines and adhered to the PRISMA (Preferred Reporting Items for Systematic Reviews and Meta-Analyses) 2020 statement. The research was performed through the databases PubMed, Scopus, and Web of Science using MeSH (Medical Subject Headings) terms for MEDLINE (PubMed), while equivalent free-text terms were applied to Scopus and Web of Science. The initial database search was performed on 20 May 2024. Studies published from 2014 to 2024 focusing on laser therapy for oral vascular lesions were included. Data quality was assessed using NHLBI and ROBINS-I V2 tools. **Results**: Of the 139 articles identified, 11 met inclusion criteria, assessing Nd:YAG, diode, Er,Cr:YSGG, and CO_2_ lasers. The Nd:YAG laser was effective for deep vascular lesions with strong thermal effects. The diode laser provided excellent coagulation and minimal postoperative discomfort. The Er,Cr:YSGG laser offered faster healing and better cosmetic results. The CO_2_ laser showed effective results with low recurrence rates. Most studies reported reduced bleeding, pain, and recovery time following laser treatment. **Conclusions**: Laser therapy, particularly Nd:YAG, diode, and CO_2_ lasers, offers a safe, effective alternative for oral vascular malformations, providing improved outcomes and fewer complications. Future studies should include larger sample sizes and comparisons with traditional therapies.

## 1. Introduction

Vascular anomalies, and more specifically vascular malformations (VMs), represent a common occurrence in the oral and maxillofacial regions, notably affecting the lips, tongue, and buccal mucosa [1]. These anomalies can be congenital and frequently become more pronounced over time [2]. Unlike hemangiomas, which generally undergo spontaneous regression during early childhood, VMs persist throughout life and tend to grow slowly, potentially increasing in size as a person ages [2].

These malformations are categorized according to the type of vessels involved, such as capillaries, veins, and lymphatic vessels [2]. Additionally, they are classified based on blood flow characteristics into low-flow and high-flow lesions [2]. Low-flow VMs, including venous lakes (VLs) and vascular venous malformations (VeMs), are particularly prevalent in the oral cavity and are often treated for aesthetic reasons, or to alleviate recurrent bleeding or functional impairment [1].

Over the past several decades, a variety of treatment modalities have been developed for managing vascular malformations, ranging from surgical resection and sclerotherapy to more conservative approaches such as laser therapy [3]. The development and refinement of laser technology have provided clinicians with a minimally invasive treatment option that offers promising results for many patients, particularly those with low-flow lesions like capillary and VeMs [3].

Various laser systems have been employed in the treatment of VMs, including the neodymium-doped yttrium aluminum garnet (Nd:YAG) laser; potassium titanyl phosphate (KTP) laser; diode lasers; flashlamp-pumped pulsed dye laser (FPDL); erbium, chromium/yttrium–scandium–gallium–garnet (Er,Cr:YSGG) laser, and carbon dioxide (CO_2_) [3,4]. These lasers are valued for their ability to selectively target and coagulate blood vessels while minimizing damage to the surrounding tissues, making them ideal for treating sensitive areas like the oral cavity [3,4].

Among the available laser systems, the Nd:YAG laser has gained particular attention due to its deep tissue penetration and high absorption by hemoglobin, rendering it especially effective for treating deeper vascular lesions [2]. Its precision and ability to coagulate blood vessels at greater depths without causing significant harm to the surface tissue make it an excellent choice for managing vascular malformations that extend into the deeper layers of the mucosa or skin [2].

Laser therapy has become an integral part of the therapeutic arsenal for vascular malformations, offering several advantages over traditional surgical methods [3]. It not only provides a more precise and less invasive treatment option but also allows for better cosmetic outcomes, particularly in cases involving superficial and aesthetically sensitive regions such as the lips and face [3]. The ability of laser systems to specifically target abnormal vessels while preserving adjacent tissues has resulted in higher patient satisfaction and improved long-term outcomes [3].

However, the management of vascular malformations, particularly in the oral region, remains complex [1]. There is currently no universally accepted treatment protocol, as the optimal approach varies depending on factors such as the size, depth, and location of the lesion, as well as individual patient characteristics [1]. Despite these challenges, the use of laser technology continues to advance, with ongoing research focusing on optimizing treatment parameters, improving long-term aesthetic results, and minimizing the potential for recurrence [5].

While studies on therapies for vascular malformations in the literature are numerous, it has not been extensively investigated which therapy is most effective, and the advantages and disadvantages for the resolution of these lesions. Additionally, the major reviews published in the literature present different issues: they do not specifically address the oral cavity [6,7,8], they are not systematic reviews [6,7,8], they are limited to one type of injury [6], or they are focused on oral cavity pathologies in general, rather than specifically on vascular malformations [9] and are focused on a pediatric population [9], while our review specifically includes studies related to any age group.

Therefore, this article aims to conduct a systematic review to assess the effectiveness of laser therapy in the treatment of oral VMs and to analyze the main advantages and disadvantages associated with different laser systems—including Nd:YAG, diode, Er,Cr:YSGG, and CO_2_ lasers—based on clinical outcomes, safety, and, where reported, aesthetic considerations. Although this review primarily focuses on comparing different laser systems, we also present descriptive data from studies that included comparisons between laser therapy and conventional treatments.

## 2. Materials and Methods

### 2.1. Study Protocol and Registration

The current review protocol has been officially registered on the PROSPERO platform (CRD42024553896), available online: https://www.crd.york.ac.uk/PROSPERO/view/CRD42024553896 (accessed on 27 May 2025).

### 2.2. Focused Questions

What are the clinical effectiveness, safety, and aesthetic outcomes of different laser systems used in the treatment of oral vascular malformations?

### 2.3. Search Strategy

A three-step search strategy was conducted in accordance with the methodology outlined by the Joanna Briggs Institute (JBI) for systematic reviews. The systematic search of PubMed, Scopus, and Web of Science was independently conducted by F.P. and A.G.

Initially, a preliminary and limited search was performed within these databases to identify relevant keywords and terms for developing a comprehensive search strategy. In the next step, terminology identified from the selected articles was used to refine and expand the search strategy. Lastly, the reference lists of all included articles were reviewed to locate any additional relevant studies [10].

The PICO framework was used to formulate the research question and to design the search strategy as explained in Table 1. A literature search was carried out in PubMed (MEDLINE), Scopus, and Web of Science, centered on the following three elements: population (participants of all ages presenting with oral cavity VMs), concept (evidence from clinical trials related to laser therapy effectiveness in the treatment of oral vascular lesions), and context (no restrictions regarding cultural factors or study settings were applied in this review). Scrutiny of study abstracts investigating the effectiveness, advantages, and disadvantages of using a laser for the treatment of VMs in the oral cavity.

Since PubMed, Scopus, and Web of Science (WoS) have different indexing systems, the search strategy was adapted accordingly. In PubMed, MeSH terms were used to enhance search precision. However, as WoS and Scopus do not support MeSH terms, an alternative approach was applied, replacing MeSH terms with equivalent free-text keywords identified through exploratory searches and structured using Boolean operators (AND, OR). This allowed for a consistent methodology across all databases.

Throughout this comprehensive literature review, adherence was maintained to the preferred reporting items for systematic reviews (PRISMA) consensus, as depicted in Table S1 (Supplementary Materials) [11]. The complete search strategy for all databases is reported in Table S2 (Supplementary Materials).

### 2.4. Eligibility Criteria

This review was based on the inclusion criteria and the exclusion criteria summarized in Table 2. VMs in children often differ from those in adults in terms of natural history, biological behavior, and treatment outcomes. Moreover, pediatric vascular lesions may require different laser parameters, and the response to laser therapy can be influenced by factors such as tissue characteristics, lesion growth patterns, and healing mechanisms.

Including only studies that examined adult or mixed-age populations allowed for a more consistent comparison of treatment effectiveness and safety.

In this systematic review, the primary focus was to compare the clinical effectiveness, safety, and aesthetic outcomes among different laser systems used for the treatment of oral vascular malformations. However, where available, we also included data from studies that compared laser therapy with conventional treatments, such as sclerotherapy or surgical excision, as additional background information. These comparisons were presented descriptively, without affecting the primary aim of the review.

### 2.5. Study Selection Process

A literature search was conducted using three electronic databases: PubMed (MEDLINE), Scopus, and Web of Science (WoS). Given the differences in indexing systems among these platforms, the search strategy was adjusted accordingly. The initial database search was performed on 20 May 2024. No additional searches were conducted prior to the submission of the final manuscript. The detailed search strategy is provided in Supplementary Materials, Table S2.

No specific software was used for the screening process. Articles were selected manually in two steps by two blinded investigators according to the previously selected eligibility criteria. M.B. and F.P. screened titles and abstracts, while M.P. and M.B. independently assessed the full texts for eligibility. Any disparities that emerged during the review were resolved through discussion with F.S.

The initial phase of screening involved the assessment of article titles and abstracts, excluding articles published in non-English languages, book chapters, narrative, systematic, or meta-analysis reviews, and not-pertinent studies with our search. Next, the selected articles were subjected to an in-depth assessment through full-text review. The findings were systematically documented, and comparable studies fulfilling the predefined inclusion criteria were identified and included in this review. A full list of excluded studies with reasons is provided in Supplementary Materials Table S3.

### 2.6. Data Extraction

After the screening process, the most relevant data on the advantages and disadvantages of using laser treatment for VMs in the oral cavity were extracted from each included study and entered into an Excel spreadsheet data extraction tool.

Data extraction was independently carried out by M.B., F.P., and M.P. verifying the accuracy of the extracted information. This tool was prepared in advance by the two lead authors. Data extraction was independently completed by the reviewers using a customized extraction form. The data extraction protocol was tested on the first five articles to ensure consistency in the extraction process. Any necessary changes to the data extraction form were made in consultation with the two lead authors and documented.

Since this systematic review includes only studies on human subjects, information on interventions and comparisons (if applicable) was extracted using the Template for Intervention Description and Replication (TIDieR) checklist items to provide a comprehensive summary of the clinical efficacy and safety of laser treatment for oral VMs. The TIDieR includes the following items: short name, why (rationale; objectives of the study; theory), what (materials; behavior change techniques; intervention and comparison group procedures), who provided the intervention, how (mode of delivery, e.g., online, face-to-face, group interventions), where (country; place of intervention, e.g., community, hospital, research laboratory), when and how much (intervention and comparison group schedule, duration, intensity, or dose of intervention), adaptation, modifications, and how well (adherence, fidelity). The main outcome measures included lesion size reduction, recurrence rate, patient-reported symptoms (e.g., bleeding, pain), and aesthetic satisfaction.

### 2.7. Quality Assessment

This review was performed by evaluating the risk of bias (RoB) by conducting a qualitative analysis of the clinical studies via the National Heart, Lung, and Blood Institute (NHLBI) Quality Assessment of Controlled Intervention Studies [12]. The RoB was independently assessed by M.B. and F.P. using the Risk Of Bias in the Non-Randomized Studies of Interventions (ROBINS-I) Version 2 (V2) tool (version 22 November 2024) [13]. Any discrepancies were resolved through consultation with M.P., C.D.T., and F.S. This method enabled a thorough and structured evaluation of study quality and potential biases, aiming to determine the trustworthiness and validity of the findings.

### 2.8. Data Synthesis and Analysis

The results were synthesized using a descriptive approach, considering the heterogeneity in study designs, laser parameters, and outcome measures among the included studies. Statistical and descriptive analyses were performed by M.P. and M.B.

Data were extracted and systematically organized into structured tables to facilitate qualitative comparisons of laser types, treatment efficacy, safety profiles, and aesthetic outcomes. Key findings were categorized based on laser wavelength and treatment modality to identify patterns in effectiveness, recurrence rates, and adverse effects. Given the variability in study methodologies and the lack of standardized outcome measures, a meta-analysis was not feasible. Whenever possible, pooled descriptive statistics (e.g., mean success rates and frequency of adverse effects) were reported to provide a comprehensive overview of treatment effectiveness.

A meta-analysis was not conducted due to significant clinical and methodological heterogeneity across the included studies. Differences in laser types, wavelengths, treatment protocols, outcome measures, and follow-up durations made statistical pooling inappropriate. In addition to differences in laser type and protocols, the included studies varied in terms of population characteristics and outcome definitions. Patient age ranged from pediatric to older adult populations, with some studies including only adults, while others reported mixed-age cohorts. The anatomical location and size of vascular malformations were inconsistently reported, further contributing to clinical variability. Moreover, outcome measures varied considerably: while some studies reported quantitative outcomes such as lesion size reduction in millimeters or percentage improvement, others used subjective scales (e.g., patient satisfaction or aesthetic outcome rated by clinicians) or did not define specific thresholds for success. Follow-up periods also ranged widely, from weeks to several years, affecting the comparability of recurrence and long-term efficacy data.

The synthesis focused on comparing treatment outcomes reported across the included studies, with results stratified by laser type, anatomical site, lesion characteristics, and patient demographics. When comparative data were available, the findings were analyzed concerning alternative treatments (e.g., surgery or sclerotherapy) to provide a thorough evaluation of the outcomes of laser therapy. The completeness of intervention reporting was evaluated using the TIDieR checklist.

## 3. Results

The initial database search yielded 139 records published between 2014 and 2024. Of these, 118 were excluded before screening for the following reasons: 31 were narrative reviews, scoping reviews, systematic or meta-analytic reviews, case series, or case reports; 10 were published in languages other than English; 46 were duplicate records; and 31 were not pertinent to the research question. After this preliminary step, 21 records were screened based on title and abstract. All 21 full-text articles were retrieved and assessed for eligibility. Of these, ten studies were excluded for the following specific reasons: four studies focused exclusively on pediatric patients, whereas the aim of this review was to evaluate laser therapy in adult or mixed-age populations to ensure homogeneity in lesion characteristics and treatment response; two studies investigated vascular lesions located outside the oral mucosa, such as those involving only the skin or lips, which fell outside the defined anatomical scope of the review; and four studies assessed therapeutic approaches that did not involve laser-based treatment, such as sclerotherapy or surgical excision, and were therefore not aligned with the intervention criteria. Ultimately, 11 studies fulfilled all inclusion criteria and were included in the final qualitative synthesis.

**Figure 1:**
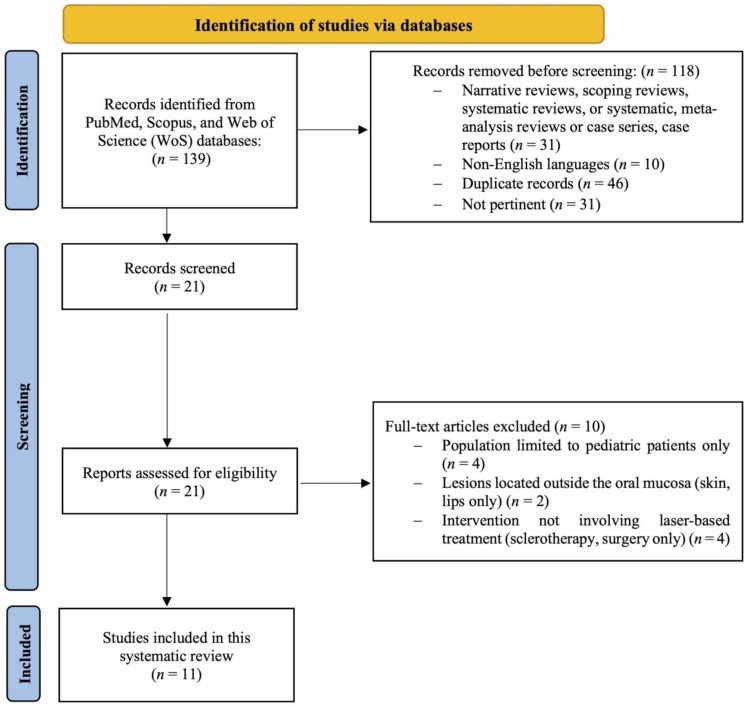
Flowchart of the systematic review procedure.

Figure 1 illustrates the PRISMA 2020 flow diagram. Table S3 (Supplementary Materials) displays the research papers not considered in this analysis and the explanations for their exclusion [14,15,16,17,18,19,20,21,22,23].

Among the studies included in this systematic review, six were retrospective observational studies, and five were prospective observational studies [1,2,3,4,5,24,25,26,27,28,29].

### 3.1. Risk of Bias

The assessment of bias risk in the articles included in this review was conducted using the ROBINS-I V2 tool (version 22 November 2024) [13]. The ROBINS-I tool evaluates seven domains, including confounding, selection bias, classification of interventions, deviations from intended interventions, missing data, outcome measurement, and selection of the reported result. Most studies were rated as having a moderate to serious risk of bias, especially in the domains of confounding and outcome measurement. Detailed assessments are available in Supplementary Tables S4–S6. The criteria for judging the RoB in the ROBINS-I V2 assessment tool [13] are outlined in Table S4 (Supplementary Materials). The outcomes of this assessment are shown in Table 3 and Table S5 (Supplementary Materials). The RoB in the ROBINS-I V2 [13] was analyzed by two blinded authors to ensure the reliability and accuracy of the evaluations. The analysis indicates a “low RoB” in the research by Abukawa et al. [24], Shivhare et al. [27], and Gobbo et al. [1], and a “moderate RoB” in the research by Asai et al. [2], Bacci et al. [4], Myazaki et al. [25], Cadavid et al. [3], Limongelli et al. [26], Nammour et al. [5], Bardhoshi et al. [28], and Gobbo et al. [29].

Studies classified as “moderate RoB”, although they handled missing data appropriately, showed shortcomings in D1 (Bacci et al. [4], Myazaki et al. [25], Limongelli et al. [26], Heimlich et al. [29]), in D7 (Nammour et al. [5]), and in D1 and D7 (Asai et al. [2], Cadavid et al. [3] and Bardhoshi et al. [28]), particularly regarding transparency in the management of the intervention and the criteria used for the selection of outcomes.

Finally, studies classified as “low RoB” such as Abukawa et al. [24], Shivhare et al. [27], and Gobbo et al. [1] demonstrated rigorous and transparent methodological practices in all areas evaluated, ensuring the reliability and validity of the results.

Table 4 displays the baseline features of the patients and control group evaluated in this systematic review, the characteristics of lesions and intervention, the number of irradiation treatments, and the number of lesions with complete healing.

Table 5 offers a comprehensive summary of the findings from the studies incorporated in this review. The table reports details such as study design and objectives, sample analysis, biomarker type, comparisons made between the two groups, and the main conclusions reported by the authors of each study.

Table 6 summarizes the main laser parameters reported in the reviewed studies.

A summary comparison of clinical outcomes across the included studies is provided in Table 7.

Table S6 shows the NHLBI Quality Assessment Tool applied to the case–control studies (Supplementary Materials). NHLBI Quality Assessment Tool for Observational Cohort and Cross-Sectional Studies is presented in Table S7 (Supplementary Materials).

### 3.2. Results of Syntheses

#### 3.2.1. Nd:YAG Laser for Deep Lesions

Several studies assessed the therapeutic effect of the Nd:YAG laser, particularly for deep VMs. Asai et al. (2014) [2] treated 67 patients and reported that the Nd:YAG laser was highly effective and minimally invasive, with no significant complications. Most lesions were smaller than 3 cm and located on the lips, buccal mucosa, and tongue. Deeper lesions required multiple treatment sessions to achieve satisfactory results. Cadavid et al. (2018) [3] analyzed 93 patients with VMs in the oral and perioral areas and confirmed the high efficacy of Nd:YAG laser photocoagulation, with most cases resolving without complications. However, deeper lesions often require multiple sessions for complete resolution.

#### 3.2.2. Diode Laser for Low-Flow Malformations

The diode laser has been extensively studied for treating low-flow VMs and VLs. Bacci et al. (2018) [4] treated 59 patients using an 830 nm diode laser and observed a significant lesion size reduction within 30 days post-treatment. Complete lesion resolution was achieved in 48 out of 59 patients at the one-year follow-up. The diode laser was associated with shorter operative times and fewer postoperative complications compared to scalpel surgery, though larger lesions (>10 mm) often required multiple sessions. Limongelli et al. (2019) [26] examined 158 lesions treated with a combination of transmucosal and cutaneous photocoagulation using a diode laser. All lesions fully healed at the one-year follow-up, and optimized laser settings significantly reduced the number of required applications.

#### 3.2.3. KTP Laser for Small VMs

Abukawa et al. (2017) [24] investigated the use of a potassium titanyl phosphate (KTP) laser in 26 patients with slow-flow VMs of the oral mucosa. The study found that lesions smaller than 30 mm responded effectively, particularly venous and capillary malformations. However, buccal mucosal lesions require careful monitoring due to a higher risk of complications such as necrosis, especially at higher energy levels.

#### 3.2.4. Er,Cr:YSGG and CO_2_ Lasers for Scar Reduction

Nammour et al. (2020) [5] compared multiple laser therapies, including Nd:YAG, diode (980 nm), Er,Cr:YSGG, and CO_2_ lasers, in 143 patients with congenital hemangiomas and VMs. Scar quality improved across all laser types, but the Er,Cr:YSGG, and CO_2_ lasers provided superior results at two weeks, with Er,Cr:YSGG achieving the highest patient satisfaction rates. Heimlich et al. (2024) [29] explored the efficacy of diode laser therapy with forced dehydration and induced photocoagulation in 47 patients showing that most patients achieved complete clinical healing after a single session, with minimal adverse effects and a low recurrence rate.

#### 3.2.5. Laser Forced Dehydration (LFD) for Low-Flow Lesions

Gobbo et al. (2024) [1] investigated the effectiveness of laser-forced dehydration (LFD) in treating low-flow vascular lesions. The study found that LFD resulted in complete lesion regression in all patients, with no significant side effects. Only one patient required a second session, and no recurrences were observed at six months or one year. This technique was effective, painless, and often required no local anesthesia, making it a valuable option for treating OVA.

#### 3.2.6. Descriptive Data from Comparative Studies: Laser vs. Other Treatments

Although this review primarily focuses on comparing different laser systems, some included studies also reported comparisons between laser therapy and conventional treatments, such as sclerotherapy or surgical excision. The findings from these studies are presented here for descriptive purposes only and were not included in the primary comparative analysis.

Shivhare et al. (2022) [27] conducted a comparative study between diode laser therapy and sclerotherapy in 40 patients. The laser group reported fewer side effects and significantly lower postoperative pain compared to the sclerotherapy group, though recurrence rates were higher in laser-treated patients. Bardhoshi et al. (2022) [28] compared the efficacy of diode laser and cold scalpel treatment for venous lip lesions in 60 patients. The 980 nm diode laser demonstrated high efficacy, with rapid healing and minimal complications. Laser treatment was well-accepted across all age groups, with patients experiencing minimal bleeding, swelling, and scarring.

## 4. Discussion

VMs are recognized as developmental anomalies from the embryonic stage, affecting arteries, veins, or capillaries [3]. Present at birth, they grow with the patient and do not regress on their own [1,3]. These malformations frequently occur in the head and neck regions, causing significant discomfort due to their aesthetic impact and the challenges involved in their removal [1,3]. Besides cosmetic issues, they can lead to bleeding, infections, blockages, pain, ulcers, and sometimes the destruction of crucial structures [3].

Management strategies for benign lesions comprise surgical excision, systemic corticosteroids, embolization, cryotherapy, interferon-α, radiotherapy, and sclerotherapy [1]. Typically, systemic corticosteroids and interferon-α are advised for larger lesions, especially congenital hemangiomas, whereas embolization is considered more appropriate for extensive VMs [1]. The development of new technologies has made laser treatment the most widely accepted approach for these lesions [1]. Over time, various devices have been introduced, such as semiconductor lasers, 514 nm argon, 532 nm KTP, 585 nm FPDL, 755 nm alexandrite, 810–940 nm diode, 1064 nm Nd:YAG, Er,Cs:YSGG, and 10,600 nm CO_2_ lasers [1,3]. In this systematic review, we compare the different types of lasers that are most used and have proven to be most effective for the treatment of vascular lesions in the oral cavity.

It is important to highlight that substantial variability was observed across the included studies regarding laser parameters such as wavelength, power output, emission mode (continuous or pulsed), and number of sessions. These differences may significantly influence treatment outcomes, and the lack of standardization complicates direct comparisons.

### 4.1. Nd:YAG Laser

According to the literature, treatment using the Nd:YAG laser for VMs is highly recommended due to its strong affinity for hemoglobin and its ability to generate heat during the procedure. This allows for it to selectively destroy the targeted tissue without harming the surrounding areas [3].

The non-invasive approach of transmucosal photothermocoagulation with the Nd:YAG laser focuses on targeting chromophores present within vascular lesions, particularly hemoglobin. These chromophores absorb the laser energy, which is subsequently transformed into heat and transferred to the walls of the blood vessels. This process causes coagulation and closure of the vessels, ultimately leading to the thrombosis of the blood vessels [5].

In a retrospective observational study conducted by Asai et al. [2], 67 patients with 69 oral VMs in total were treated with Nd:YAG laser, 1064 wavelength, at an output of 8–15 W [2]. The Nd:YAG laser beam penetrated approximately 6 mm into the tissue, enabling therapeutic irradiation to be effectively completed in a single session for most cases (62 out of 69) [2]. Consequently, the Nd:YAG laser exhibits a strong thermal effect in deep tissues and demonstrates powerful coagulation and hemostasis capabilities [2]. Furthermore, the Nd:YAG laser has proven effective for lesions of both small and large diameters [2]. In the study by Asai et al. [4], patients with lesions smaller than 15 mm were deemed treatable using the Nd:YAG laser [2]. For lesions 15 mm or larger, all but one case, which required seven irradiation sessions, were successfully treated using external Nd:YAG laser irradiation alone, or in combination with another laser or surgical scalpel [2].

Miyazaki et al. [25], in a 2018 clinical study, use the leopard technique, which consists of delivering Nd:YAG laser energy in a “spot” mode, with each spot realized with single-wave irradiation. For extensive lesions thicker than 1 cm or those with a dense mucosal covering, laser therapy (LT) was combined with intralesional photocoagulation (ILP) in continuous mode with power settings ranging from 10 to 15 W, with or without ultrasound (US) guidance [25]. All treated lesions showed a size reduction, with no further enlargement observed after the initial edema subsided [25].

It has been seen in the literature that despite the proven efficacy of Nd:YAG, multiple operating sessions are sometimes necessary [5]. In particular, in the study by Nammour et al. [5], it was seen that lesions treated with Nd:YAG had a recurrence rate of 8% ± 0.9% Nd:YAG, which means that patients required additional treatment sessions.

Nevertheless, in all compared studies, after treatment, no patients experienced complications and reached clinically and aesthetically satisfactory results [3]. These results were primarily observed in the in the study conducted by Cadavid et al. [3] in 93 patients treated with the Nd:YAG laser. The outcomes obtained suggest that this type of laser is effective and safe for treating oral and perioral VMs [3].

Although future comparative studies are needed to confirm its superiority over other techniques, the Nd:YAG laser is currently a successful therapy due to its efficacy, safety, and ease of use, with minimal postoperative complications.

### 4.2. Diode Laser

In recent years, the use of high-power diode lasers in managing oral vascular anomalies (OVA) has significantly increased and has proven effective for treating superficial vascular lesions, as demonstrated in various studies. Several authors have reported comparable clinical outcomes with excellent healing, even when different laser parameters were used [29]. The diode laser offers several advantages, including excellent coagulation properties, absence of postoperative bleeding and pain, and effective wound healing [29]. As a result, patients benefit from improved postoperative appearance, with reduced edema, bleeding, infection, and discomfort, leading to a decreased need for analgesics.

These outcomes were confirmed by Heimlich et al. [29], who used pulsed diode laser mode for OVA. The number of sessions ranged from one to four, with 83% of patients requiring only a single treatment. While diode laser treatment generally results in complete lesion excision, in some cases, multiple sessions may be necessary [29]. Similarly, Bacci et al. [4] reported that although not all malformations were completely resolved after a single procedure, 74.6% of cases showed excellent lesion volume reduction one month after treatment. Importantly, diode laser treatment can be safely repeated in patients with residual lesions, ultimately achieving full resolution [4].

A patient-specific approach based on lesion size and depth has also been shown to reduce the number of sessions required. In the study by Limongelli et al. [26], lesions were categorized by surface size (<1 cm, 1–3 cm, >3 cm) and depth (≤5 mm or >5 mm) and treated with an 800 ± 10 nm diode laser. Their findings indicated that adjusting laser settings according to these parameters significantly optimized treatment outcomes and reduced the number of applications. Furthermore, combining diode laser treatment with intraoperative tissue cooling and postoperative regenerative gel application improved tolerance, minimized complications, and accelerated healing [26].

Gobbo et al. [1] further supported the efficacy of diode laser therapy in a recent study involving 30 patients. They evaluated postoperative parameters such as pain, bleeding, scar formation, and need for retreatment. Pain and scar formation were reported in only one case, while bleeding occurred in another. A multiwavelength diode laser (810–830 nm) was used in the study and was found to provide safe, painless, and effective treatment across all evaluated parameters [1].

### 4.3. Er,Cr:YSGG Laser

Erbium, chromium-doped yttrium, scandium, gallium, and garnet (Er,Cr:YSGG) lasers were introduced in 1997 for clinical dentistry [30]. Erbium, a rare earth element, is embedded in a host crystal, with the illumination occurring in the Er^3+^ ion [30]. The host crystals are made of yttrium, aluminum, and garnet (Y3A5O12) and yttrium, scandium, gallium, and garnet (Y3Sc2Ga3O12) [30]. Studies show that erbium lasers promote faster healing due to their low thermal effect and reduce the need for antibiotics [30].

The research conducted by Nammour et al. [5] shows the results after the treatment of the lesion with different types of laser devices and demonstrates the prerogative of good healing after the use of an Er,Cr:YSGG laser. At the two-week follow-up, both Er,Cr:YSGG and CO_2_ lasers demonstrated significantly superior scar quality compared to Nd:YAG and diode lasers [5]. Furthermore, the Er,Cr:YSGG laser yielded the highest patient satisfaction scores at the same time point [5].

### 4.4. Carbon Dioxide Laser (CO_2_)

The CO_2_ laser was one of the earliest lasers used for soft tissue removal [31]. Due to its strong affinity for water, it excels at removing, vaporizing, and coagulating soft tissues [31]. In minor oral surgery, CO_2_ LT has demonstrated several advantages [31]. Healing following treatment of vascular lesions with CO_2_ laser appears to be effective and with a low recurrence rate [31]. In the study by Nammour et al. [5], a CO_2_ laser with a wavelength of 10,600 nm was employed in a defocused (non-contact) mode, operating in continuous wave (CW) with an output power of 1 W. The surgical procedure was deemed complete upon confirmation of hemostasis, achieved through gentle pressure applied to the surgical site. The wound was left to heal by secondary intention, and no sutures were placed [5]. After the treatment, no recurrence was observed with CO_2_ laser over 12 months [5].

### 4.5. Potassium Titanyl Phosphate Laser (KTP)

Since its introduction in 1980, the KTP laser has been used to treat vascular lesions. Its selective absorption by hemoglobin makes it ideal for addressing vascular anomalies. However, the KTP laser’s coagulation effect is restricted to a depth of 1 to 2 mm [24]. In a study by Abukawa et al. [24], 26 patients with a total of 38 lesions (average size 13.5–7.7 mm) were treated with a KTP laser, at a constant power of 2 W. Treatment results indicated that lesions smaller than 30 mm achieved cure and regression, while those larger did not respond [24]. Lesions on the tongue and lips had higher cure rates compared to other areas. Using less than 400 joules of energy, 68% of lesions were effectively treated [24]. Higher complication rates, including necrosis, were observed in buccal mucosal lesions and with high-energy treatments [24].

### 4.6. Laser Versus Cold Scalpel and Sclerotherapy

Some of the included studies provided additional comparisons between laser therapy and conventional treatments, such as surgical excision or sclerotherapy [27,28]. While these findings offer background information, they were not included in the primary comparative analysis, which focused exclusively on the differences among laser systems. Therefore, no definitive conclusions regarding the comparative effectiveness between laser therapy and conventional treatments can be drawn from this review.

Sclerotherapy is a technique used to eliminate small vessels, varicose veins, and vascular anomalies by injecting a biomaterial known as a sclerosant [27]. The sclerosing agent induces noticeable tissue irritation, leading to minimal thrombosis, endothelial damage, and localized inflammation and tissue necrosis [27]. The inflammation and necrosis result in fibrosis and contracture, which ultimately cause the lesion to disappear [27]. Shivhare et al. [27], in a 2022 study, compared two groups, one treated with diode laser and the other with sclerotherapy. The diode laser offers fewer side effects and greater patient comfort compared to sclerotherapy [27]. For small or cosmetically sensitive lesions, such as those on the palate, the laser is preferred due to its minimal adverse effects, while sclerotherapy is more suitable for larger lesions [27]. Moreover, one of the most debated topics in this field is the choice between a laser and a scalpel [28]. Among the evaluated studies, Bardhoshi et al. [28] treated 30 patients with a 980 nm diode laser and other 30 patients with a cold scalpel, demonstrating that the clinical application of a 980 nm diode laser for managing vascular lesions of the lip has beneficial effects due to its good absorption in hemoglobin. Laser treatment is quick, well-accepted across all age groups, and has fast healing [28]. It has been observed that following surgical excision, the use of sutures is necessary to control bleeding and wound healing. Furthermore, 22 out of 30 patients reported pain, in contrast to laser-treated patients who reported no pain [28].

### 4.7. Limitations and Future Studies

This study has certain limitations that should be taken into account when interpreting the findings. Primarily, the modest sample size may restrict the applicability of the results, highlighting the importance of conducting future research with larger cohorts to enhance statistical reliability. Moreover, all included studies were observational in design (either prospective or retrospective), which inherently limits the level of evidence. The lack of randomized controlled trials (RCTs) weakens the robustness of the conclusions and raises the potential for selection bias and confounding variables. Future research should prioritize well-designed RCTs comparing laser therapy with standard treatments. Additionally, the lack of a randomized control group limits the capacity to reach definitive conclusions regarding the effectiveness of laser therapy in comparison to alternative treatments, emphasizing the need for controlled groups in future clinical research.

Another important limitation is the exclusion of studies focused exclusively on pediatric populations. This choice was made to ensure greater homogeneity in lesion characteristics and treatment responses, as VMs in children often differ from those in adults in terms of natural history, biological behavior, and therapeutic response. Furthermore, pediatric vascular lesions may require different laser parameters, and the response to laser therapy can be influenced by factors such as tissue characteristics, lesion growth patterns, and healing mechanisms. Including only studies that examined adult or mixed-age populations allowed for a more consistent comparison of treatment effectiveness and safety. However, further studies are needed to explore the specific effectiveness and safety of laser therapy in pediatric populations, taking into account the distinct biological and clinical characteristics of this group.

The heterogeneity of the patient characteristics included in the studies, such as age and comorbidities, may have also influenced the results, suggesting the need for future studies focusing on more homogeneous cohorts. Moreover, the short follow-up period does not allow for a comprehensive assessment of the durability of treatment effects. Long-term studies will be essential to examine treatment persistence and recurrence rates.

Another limitation is the lack of direct comparison between laser treatment and other techniques, such as surgery or sclerotherapy, which restricts our understanding of the relative advantages of laser therapy over alternative options.

Future studies should include standardized treatment protocols and clearly defined comparator groups to enable a more accurate evaluation of the efficacy, safety, and cost-effectiveness of laser therapy in the management of oral vascular malformations (VMs). Additionally, adopting standardized outcome measures and incorporating long-term follow-up will be essential to assess the persistence of treatment effects and the potential for recurrence over time.

Particular emphasis should be placed on patient-centered endpoints, such as functional outcomes, aesthetic satisfaction, and quality of life, which are critical for refining therapeutic strategies and informing clinical decision-making in a meaningful and individualized manner.

Due to the substantial heterogeneity in study design, laser parameters, outcome definitions, and patient populations—as well as the exclusive inclusion of non-randomized studies—it was not appropriate to apply the Grading of Recommendations, Assessment, Development, and Evaluation (GRADE) framework in this review. The lack of homogeneity in effect estimates and the absence of consistent direct comparisons further limited the applicability of GRADE to rate the certainty of evidence across outcomes. Future high-quality research, particularly randomized controlled trials with standardized methodologies and outcome reporting, will be necessary to support GRADE-based assessments of the quality and strength of evidence regarding laser therapy for oral VMs.

Despite these limitations, the structured synthesis of the available evidence offers valuable insights into the effectiveness and safety of laser therapy. Future research should aim to refine laser protocols, optimize energy settings for different lesion types, and assess long-term clinical and aesthetic outcomes to establish evidence-based guidelines for the management of oral VMs.

## 5. Conclusions

This systematic review confirms the clinical effectiveness of laser therapy for oral vascular malformations, demonstrating positive results in terms of lesion reduction, rapid healing, and aesthetic improvement. Among the various laser systems analyzed, Nd:YAG, diode laser, and CO_2_ laser appear to be safe and effective options, with low rates of postoperative complications and a reduced need for additional treatments. The findings support the use of laser therapy as a minimally invasive approach for managing these lesions, with the choice of laser system potentially tailored to the specific characteristics of the vascular malformation.

Although the primary focus of this review was the comparison among different laser systems, some included studies also provided descriptive comparisons between laser therapy and conventional treatments, such as surgical excision or sclerotherapy. However, these comparisons were limited, and no definitive conclusions can be drawn regarding the relative effectiveness of laser therapy versus these traditional approaches. Further high-quality comparative studies, including randomized controlled trials, are needed to strengthen the evidence base and guide clinical decision-making.

## Figures and Tables

**Table 1:** PICO framework for the systematic review on laser therapy in oral VMs.

1. Participants/Population: participants diagnosed with VMs of the oral cavity, including both adults and mixed-age populations, but excluding studies limited to pediatric patients
2. Intervention/Exposure: laser therapy for the excision of vascular malformation
3. Comparison/Control: comparison among different laser systems in the treatment of vascular lesions
4. Outcomes: clinical effectiveness, safety, and aesthetic outcomes in the treatment of vascular lesions

**Table 2:** Inclusion and exclusion criteria followed in this review.

Inclusion Criteria	Exclusion Criteria
Study design: including retrospective and prospective cohort studies, case-control studies, and randomized controlled trials	Abstracts of articles published in non-English languages
10-year time frame (from 2014 to 2024)	Duplicate studies
Human participants diagnosed with VMs of the oral cavity, including adults and mixed-age populations, but not studies exclusively focused on pediatric patients	Book chapters, narrative, systematic, meta-analysis reviews, case series, or case reports
Interventions: Laser therapy for the excision of vascular malformation	Studies focused exclusively on pediatric populations were excluded to ensure homogeneity in lesion characteristics and treatment response
Outcome: Laser effectiveness and safety in the treatment of vascular lesions	-

**Table 3:** Bias analysis using the ROBINS-I V2 tool [13] for observational studies.

Reference First Author et al. Year	A A1/A2/A3			B B1/B2		C	D1 (VA)	D2	D3	D4 (VA)	D5	D6	D7	Overall Risk-of-Bias Judgement
Gobbo et al., 2024 [1]	PN	PN	N	N (D1—VA)	/	To assess the intention-to-treat effect (the effect of assignment to an intervention strategy or comparator strategy) (D4—VA).	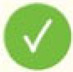	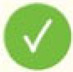	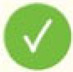	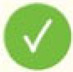	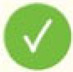	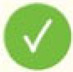	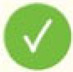	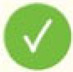
Asai et al., 2014 [2]	PN	PN	N	N (D1—VA)	/	To assess the intention-to-treat effect (the effect of assignment to an intervention strategy or comparator strategy) (D4—VA).	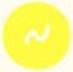	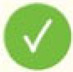	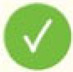	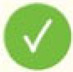	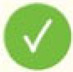	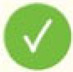	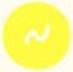	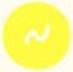
Cadavid et al., 2018 [3]	PN	PN	N	N (D1—VA)	/	To assess the intention-to-treat effect (the effect of assignment to an intervention strategy or comparator strategy) (D4—VA).	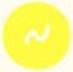	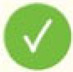	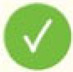	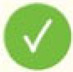	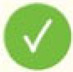	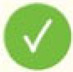	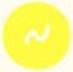	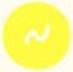
Bacci et al., 2018 [4]	PN	PN	N	N (D1—VA)	/	To assess the intention-to-treat effect (the effect of assignment to an intervention strategy or comparator strategy) (D4—VA).	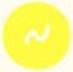	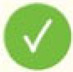	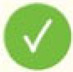	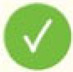	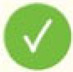	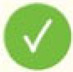	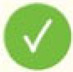	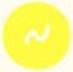
Nammour et al., 2020 [5]	PY	NA	N	N (D1—VA)	/	To assess the intention-to-treat effect (the effect of assignment to an intervention strategy or comparator strategy) (D4—VA).	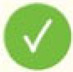	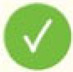	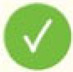	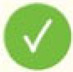	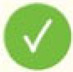	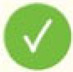	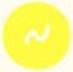	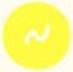
Abukawa et al., 2017 [24]	PY	NA	N	N (D1—VA)	/	To assess the intention-to-treat effect (the effect of assignment to an intervention strategy or comparator strategy) (D4—VA).	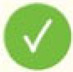	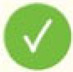	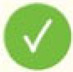	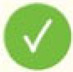	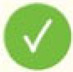	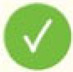	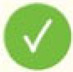	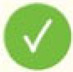
Miyazaki et al., 2018 [25]	PN	PN	N	N (D1—VA)	/	To assess the intention-to-treat effect (the effect of assignment to an intervention strategy or comparator strategy) (D4—VA).	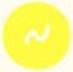	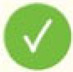	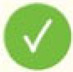	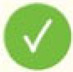	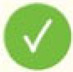	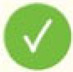	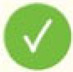	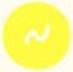
Limongelli et al., 2019 [26]	PN	PN	N	N (D1—VA)	/	To assess the intention-to-treat effect (the effect of assignment to an intervention strategy or comparator strategy) (D4—VA).	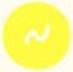	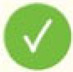	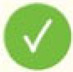	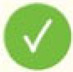	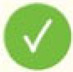	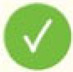	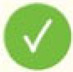	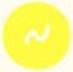
Shivhare et al., 2022 [27]	PY	NA	N	N (D1—VA)	/	To assess the intention-to-treat effect (the effect of assignment to an intervention strategy or comparator strategy) (D4—VA).	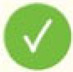	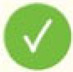	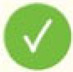	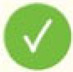	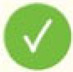	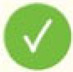	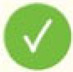	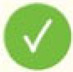
Bardhoshi et al., 2022 [28]	PN	PN	N	N (D1—VA)	/	To assess the intention-to-treat effect (the effect of assignment to an intervention strategy or comparator strategy) (D4—VA).	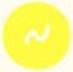	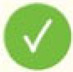	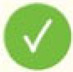	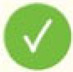	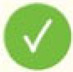	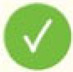	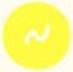	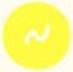
Heimlich et al., 2024 [29]	PN	PN	N	N (D1—VA)	/	To assess the intention-to-treat effect (the effect of assignment to an intervention strategy or comparator strategy) (D4—VA).	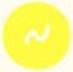	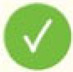	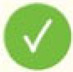	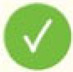	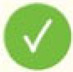	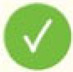	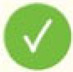	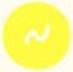

Abbreviations: A: preliminary decision on whether to proceed with the risk of bias assessment; B: specification of the analysis within the study to which the risk of bias assessment applies; C: definition of the effect of interest (e.g., intention-to-treat vs. per-protocol); D1: bias due to confounding; D2: bias in classification of interventions; D3: bias in selection of participants into the study or analysis; D4—VA: bias due to deviations from intended interventions (Variant A—effect of assignment to intervention); D5: bias due to missing data; D6: bias in outcome measurement; D7: bias in selection of the reported result; green symbol: low risk of bias; yellow symbol: moderate risk of bias; VA: Variant A (effect of assignment to intervention).

**Table 4:** Baseline characteristics of patients, lesions, and interventions included in selected studies.

References (Authors, Year, and Publication Country)	N° of Patients (N° Woman)	Mean Age ± SD and/or Range (Years)	Lesion Size (n° of Lesions)	Treated Lesion Number	Anatomical Site (n° of Lesions)	N° of Lesions with Complete Healing
Gobbo et al., 2024 Italy [1]	30 (16)	67, range 41–86	2–25 mm	30	Tongue (4), gingiva (2), lip (19), palate (1), cheek (4)	N.R.
Asai et al., 2014 Japan [2]	67 (44)	50.0, range 7–89	Group 1 <15 mm (56); Group 2 >15 mm (13)	69	Lip (33), tongue (25), buccal mucosa (6), gingiva (2), mouth floor (2), soft palate (1)	62 of 69
Cadavid et al., 2018 Brazil [3]	93 (57)	63.3, range 8–85	Most lesions were <3 cm	104	Inferior lip (44), superior lip (15), tongue (13), palate (6), jugal mucosa (22), gum (3), retromolar trigone (1)	N.R.
Bacci et al., 2018 Italy [4]	59 (28)	53, range 30–73	N.R.	59	Lip (18), tongue (15), alveolar mucosa (16), palate (7), gum (3)	48 of 59
Nammour et al., 2020 Belgium [5]	143 (81)	48, range 43–74	3–15 mm	143	N.R.	N.R.
Abukawa et al., 2017 Japan [24]	26 (17)	55.7	<9 mm (13), 10–19 mm (19), 19–29 mm (4), >30 mm (2)	38	Tongue (13), lip (11), buccal mucosa (6), gingiva (3), masseter (3), floor of the mouth (1), palate (1)	N.R
Miyazaki et al., 2018 Japan [25]	46	N.R.	N.R.	47	Tongue (17), buccal mucosa (30), gingiva (0)	36 of 47
Limongelli et al., 2019 Italy [26]	112 (52)	42, N.R.	Group A (52) <1 cm; Group B1 (28) 1–3 cm and <5 mm depth; Group B2 (16) 1–3 cm and >5 mm depth; Group C1 (42) >3 cm and <5 mm depth; Group C2 (12) >3 cm and >5 mm depth.	158	Lower lip (32), upper lip (9), tongue (41), fornix (5), palate (12), cheek (46), perioral skin (13)	100‰ (Group A); 93% (Group B1); 90% (Group B2); 88% (Group C1); 84% (Group C2)
Shivhare et al., 2022 India [27]	40 (16)	N.R.	<1 cm (15), 1–2 cm (10), 2–3 cm (8), >3 cm (7)	40	Tongue (18), buccal mucosa (11), lower labial mucosa (6), upper labial mucosa (3), palate (2)	N.R.
Bardhoshi et al., 2022 Albania [28]	60 (28)	N.R., range 10–80	N.R.	60	N.R.	N.R.
Heimlich et al., 2024 Brazil [29]	47 (17)	57.4 (±14.9), range 7–81	2–20 mm	47	Lower lip (22), upper lip (13), tongue (7), buccal mucosa (4), alveolar ridge (1)	N.R.

Abbreviations: N.R.: not reported.

**Table 5:** Summary of evidence from the studies included in this review.

Authors and Publication Year	Study Type and Objectives	Methods	Inclusion and Exclusion Criteria	Results	Conclusions
Gobbo et al., 2024 Italy [1]	Prospective observational study aimed at evaluating the effectiveness of laser forced dehydration (LFD) on low-flow vascular lesions.	Lesions were treated using LFD and followed up at 3 weeks, 6 months, and 1 year. In cases of incomplete healing at 3 weeks, an additional session was performed. Outcomes assessed included pain (NRS 0–10), analgesic requirement, bleeding, and scar formation.	Inclusion: pigmented, soft, non-pulsatile lesions with positive diascopy, accessible to laser. Exclusion: age < 18, pregnancy or breastfeeding, refusal to participate in 1-year follow-up.	Complete lesion regression was observed in all cases. One patient required a second session. No pain was reported (NRS = 0), and no analgesics were used. Minor bleeding occurred in one patient and a small scar in another. No recurrences were reported.	LFD proved to be effective and painless, often without anesthesia. Glass slides can minimize bleeding. Anatomical challenges may require multiple low-power sessions.
Asai et al., 2014 Japan [2]	Retrospective observational study assessing the clinical effectiveness of Nd:YAG laser photocoagulation for oral VMs.	Between 2004 and 2011, 67 patients were treated using 8–15 W Nd:YAG laser under local anesthesia.	Inclusion/Exclusion: N.R.	Deeper lesions required multiple sessions. External laser application alone yielded satisfactory results without clinical complications.	Nd:YAG laser was found effective and minimally invasive, with no significant adverse events.
Cadavid et al., 2018 Brazil [3]	Retrospective observational study evaluating the efficacy of Nd:YAG laser photocoagulation in treating oral and perioral VMs.	From 2006 to 2013, 93 patients were treated using 1064 nm Nd:YAG laser (400 µm fiber) under local anesthesia.	Inclusion/Exclusion: N.R.	No complications were recorded. Deep lesions required multiple treatment sessions.	Nd:YAG laser was effective and minimally invasive, with excellent aesthetic outcomes. Further studies are needed to confirm superiority over alternative methods.
Bacci et al., 2018 Italy [4]	Prospective observational study evaluating transmucosal diode laser treatment in patients with VeMs or VLs.	Fifty-nine patients with low-flow lesions were treated with 830 nm diode laser (1.6 W) and followed up at 7 days, 30 days, and 1 year. Pain was recorded daily for the first 7 days.	Exclusion: Pulsatile lesions, syndromic conditions, prior immunosuppressive therapy, extra-oral extension, or vascular tumors.	At 30 days, 52 patients showed excellent/good lesion reduction. Six required a second treatment. At 1 year, 48 achieved complete resolution; five experienced recurrence.	Diode laser is effective with reduced operative time and fewer complications. Lesions > 10 mm may require multiple sessions.
Nammour et al., 2020 Belgium [5]	Retrospective observational study evaluating scar quality, recurrence, and patient satisfaction across various laser treatments.	143 patients with congenital hemangiomas or VMs were treated with Nd:YAG, diode (980 nm), Er,Cr:YSGG, or CO_2_ lasers. Follow-up was 12 months.	Inclusion: Patients with unaesthetic VL. Exclusion: Chronic diseases, diabetes, immunosuppression, concurrent tumors.	All lasers improved scar quality at 12 months. Er,Cr:YSGG and CO_2_ lasers had better results at 2 weeks. The highest level of patient satisfaction was reported with Er,Cr:YSGG.	Multiple laser types are effective. Er,Cr:YSGG yielded the best early aesthetic outcomes.
Abukawa et al., 2017 Japan [24]	Prospective observational study aimed to determine the resolution rate of slow-flow VMs and identify risk and prognostic factors linked to successful outcomes with potassium titanyl phosphate (KTP) laser treatment.	Twenty-six patients underwent intralesional laser photocoagulation under local anesthesia, with a consistent KTP laser power of 2 watts. Outcomes were measured through clinical assessments and lesion size reduction observed on magnetic resonance imaging (MRI).	Inclusion Criteria: Patients with diagnosed venous or capillary malformations. Exclusion Criteria: Patients with hemangiomas, arteriovenous malformations, lymphatic malformations, those on anticoagulants, or those who did not undergo MRI.	Lesions treated with less than 400 joules responded effectively. However, buccal mucosal lesions and those treated with higher energy levels had a higher rate of complications like necrosis.	KTP laser therapy effectively treated slow-flow VMs smaller than 30 mm with minimal side effects.
Miyazaki et al., 2018 Japan [25]	Retrospective observational study assessing efficacy of Nd:YAG laser with multiple spot single-pulse technique.	46 patients with 47 lesions treated with 1064 nm Nd:YAG laser; 24 lesions required combined intralesional therapy.	Inclusion/Exclusion: N.R.	Satisfactory outcomes in all cases; no serious adverse events like ulceration, bleeding, or scarring.	Nd:YAG laser is a safe, less invasive option for managing oral vascular lesions.
Limongelli et al., 2019 Italy [26]	Retrospective observational study to determine optimal diode laser settings for head and neck VMs.	158 lesions categorized by size and depth, treated with 800 ± 10 nm diode laser using transmucosal, cutaneous, or intralesional photocoagulation.	Inclusion/Exclusion: N.R.	All lesions fully healed at 1 year. Larger/deeper lesions required up to 10 sessions. Mild-moderate postoperative pain occurred in 5–7% of cases.	Optimized laser parameters improved tolerance, healing time, and reduced complications.
Shivhare et al., 2022 India [27]	Prospective observational study comparing diode laser and sclerotherapy for oral VMs.	40 patients randomly assigned to diode laser (980 nm) or sclerotherapy (3% sodium tetradecyl sulfate). Outcomes assessed using chi-square test.	Inclusion: Low-flow lesions confirmed by Doppler and CT angiogram. Exclusion: High-flow lesions or systemic anomalies.	Laser group had significantly fewer side effects and less pain. Recurrence was more frequent compared to sclerotherapy.	Both treatments are effective. Laser is better tolerated but may have higher recurrence.
Bardhoshi et al., 2022 Albania [28]	Retrospective observational study comparing diode laser and cold scalpel for VLs.	60 patients treated between 2007–2012; 30 with 980 nm diode laser, 30 with scalpel surgery. Follow-up at 1, 6, and 12 months.	Inclusion/Exclusion: N.R.	Laser showed better outcomes in bleeding control, patient acceptance, and scar minimization.	Diode laser is effective across all age groups with superior hemostatic and aesthetic results.
Heimlich et al., 2024 Brazil [29]	Prospective observational study on diode laser photocoagulation (FDIP) in OVA.	47 patients treated with 808 nm diode laser (4.5 W, pulsed mode). Follow-up assessed healing rate and recurrence.	Inclusion: Esthetic/functional concerns, superficial low-flow lesions. Exclusion: Previous treatments, systemic disease, or lack of follow-up.	Complete healing in 70.5% within 30 days. Edema occurred in 66%, and recurrence in 4.2%.	FDIP is effective and safe, often requiring only a single session with minimal adverse effects.

Abbreviations: FDIP: forced dehydration with induced photocoagulation. LFD: laser forced dehydration. MRI: magnetic resonance imaging. N.R.: not reported. NRS: Numeric Rating Scale. OVA: oral vascular anomaly. VeMs: Vascular venous malformations. VL: vascular lesion. VLs: venous lakes. VMs: vascular malformations.

**Table 6:** Technical parameters and application protocols of laser devices used for the treatment of oral VMs in included studies.

Authors and Year of Publication	Laser Type	Wavelength (nm)	Power Output (W)	Emission Mode	Sessions (Number of Lesions)	Cooling/Gel Used
Gobbo et al., 2024 Italy [1]	Diode laser	445 nm 970 nm	N.R.	Pulsed	N.R.	N.R.
Asai et al., 2014 Japan [2]	Nd:YAG laser	1064 nm	8–15 W	N.R.	1 (62), 2 (5), 3 (1), 7 (1)	N.R.
Cadavid et al., 2018 Brazil [3]	Nd:YAG laser	1064 nm	3 W	Pulsed	N.R.	N.R.
Bacci et al., 2018 Italy [4]	Diode laser	830 nm	1.6 W	Continuous	1 (46), 2 (6)	Physiological solution
Nammour et al., 2020 Belgium [5]	Nd:YAG, diode, Er,Cr:YSGG, CO_2_	1064 nm, 980 nm, 2790 nm, 10,600 nm	2 W, 4 W, 0.25 W, 1 W	Pulsed: 320 μs Continuous Pulsed: 60 μs Continuous	N.R. (47) N.R. (32) N.R. (12) N.R. (52)	N.R.
Abukawa et al., 2017 Japan [24]	KTP laser	N.R.	2 W	N.R.	Average 1.1 (N.R.)	N.R.
Miyazaki et al., 2018 Japan [25]	Nd:YAG laser	1064 nm	10 W	Pulsed: 20 ms	N.R.	N.R.
Limongelli et al., 2019 Italy [26]	Diode laser	800 ± 10 nm	8 W 12 W 13 W	Pulsed: 190 ms t-on and 250 ms t-off	1(52), 2 (28), 3 (16), 5 (42), 10 (20)	Topic cryotherapy with ice packs
Shivhare et al., 2022 India [27]	Diode laser	980 nm	2–3 W	Continuous	1 session (22), 2 session (11), 3 or >3 (7)	N.R.
Bardhoshi et al., 2022 Albania [28]	Diode laser	980 nm	3 W	Continuous	N.R.	N.R.
Heimlich et al., 2024 Brazil [29]	Diode laser	800 ± 10 nm	4.5 W	Pulsed: 25 ms	1 session	N.R.

**Table 7:** Summary comparison of clinical outcomes across the included studies.

Authors and Year of Publication	Treated Lesion Number	Complete Healing	Aesthetic Outcome	Complications
Gobbo et al., 2024 Italy [1]	30	N.R.	N.R.	N.R.
Asai et al., 2014 Japan [2]	69	62/69	Good aesthetic outcomes	No major complications
Cadavid et al., 2018 Brazil [3]	104	N.R.	N.R.	Minimal side effects reported
Bacci et al., 2018 Italy [4]	59	48/59	Excellent cosmetic results	Minor discomfort
Nammour et al., 2020 Belgium [5]	143	N.R.	N.R.	No serious adverse effects
Abukawa et al., 2017 Japan [24]	38	N.R.	N.R.	No complications reported
Miyazaki et al., 2018 Japan [25]	47	36/47	High satisfaction rate	Mild postoperative pain
Limongelli et al., 2019 Italy [26]	158	Group A: 100%; Group B1: 93%; B2: 90%; C1: 88%; C2: 84%	Improved cosmetic appearance	No significant adverse events
Shivhare et al., 2022 India [27]	40	N.R.	N.R.	N.R.
Bardhoshi et al., 2022 Albania [28]	60	N.R.	N.R.	N.R.
Heimlich et al., 2024 Brazil [29]	47	N.R.	N.R.	N.R.

Abbreviations: N.R.: not reported.

## Data Availability

Upon request to the corresponding author, the data are available for use.

## Supplementary Materials

The following supporting information can be downloaded at: https://www.mdpi.com/article/10.3390/dj13090416/s1, Table S1: PRISMA 2020 Checklist [11]; Table S2: Search strategies used for each database and number of records retrieved; Table S3: Summary table of studies excluded in this systematic review [14,15,16,17,18,19,20,21,22,23]; Table S4: Criteria for judging risk of bias in ROBINS-I Version 2 (V2) tool [13]; Table S5: Assessment of the risk of bias specific to each domain of the ROBINS-I V2 tool [1,2,3,4,13,24,25,26,27,28,29]; Table S6: NHLBI Quality Assessment Tool for Case-Control Studies [2,3,5,12,25,26,28]; Table S7: NHLBI Quality Assessment Tool [12] for Observational Cohort and Cross-Sectional Studies [1,4,24,27,29].

## Abbreviations

CO_2_	Carbon dioxide
CW	Continuous wave
Er,Cr:YSGG	Erbium, Chromium: Yttrium-Scandium-Gallium-Garnet.
FDIP	Forced dehydration with induced photocoagulation
ILP	Intralesional photocoagulation
KTP	Potassium titanyl phosphate
LT	Laser therapy
Nd:YAG	Neodymium-doped Yttrium Aluminum Garnet.
NHLBI	National Heart, Lung, and Blood Institute
OVA	Oral vascular anomaly
PRISMA	Preferred Reporting Items for Systematic Reviews
RR	Relative risk
RoB	Risk of bias
ROBINS-I V2	Risk Of Bias in Non-Randomized Studies—of Interventions, Version 2
VLs	Venous lakes
VeMs	Vascular venous malformations
VMs	Vascular malformations
WoS	Web of Science
