# Supplementary material for: Laser Therapy for Vascular Malformations of the Oral Cavity: A Systematic Review
Source: Dent J (Basel). 2025 Sep 9;13(9):416. doi: 10.3390/dj13090416 (PMC12468589; doi:10.3390/dj13090416)
Supplement: Supplementary file 1 [file dentistry-13-00416-s001.zip › dentistry-3748693-supplementary.pdf]

Table S1. PRISMA 2020 Checklist [11].

Section and Topic	Item #	Checklist item	Location where item is reported
**TITLE**			
Title	1	Identify the report as a systematic review.	Page 1
**ABSTRACT**			
Abstract	2	See the PRISMA 2020 for Abstracts checklist.	Page 1-2
**INTRODUCTION**			
Rationale	3	Describe the rationale for the review in the context of existing knowledge.	Page 2-3
Objectives	4	Provide an explicit statement of the objective(s) or question(s) the review addresses.	Page 2-3
**METHODS**			
Eligibility criteria	5	Specify the inclusion and exclusion criteria for the review and how studies were grouped for the syntheses.	Page 3
Information sources	6	Specify all databases, registers, websites, organisations, reference lists and other sources searched or consulted to identify studies. Specify the date when each source was last searched or consulted.	Page 4
Search strategy	7	Present the full search strategies for all databases, registers and websites, including any filters and limits used.	Page 4
Selection process	8	Specify the methods used to decide whether a study met the inclusion criteria of the review, including how many reviewers screened each record and each report retrieved, whether they worked independently, and if applicable, details of automation tools used in the process.	Page 4
Data collection process	9	Specify the methods used to collect data from reports, including how many reviewers collected data from each report, whether they worked independently, any processes for obtaining or confirming data from study investigators, and if applicable, details of automation tools used in the process.	Page 4
Data items	10a	List and define all outcomes for which data were sought. Specify whether all results that were compatible with each outcome domain in each study were sought (e.g. for all measures, time points, analyses), and if not, the methods used to decide which results to collect.	Page 4-5
	10b	List and define all other variables for which data were sought (e.g. participant and intervention characteristics, funding sources). Describe any assumptions made about any missing or unclear information.	Page 4-5
Study risk of bias	11	Specify the methods used to assess risk of bias in the included studies, including details of the tool(s) used, how many reviewers assessed each study and whether they worked independently, and if applicable, details of	Page 6

Section and Topic	Item #	Checklist item	Location where item is reported
assessment		automation tools used in the process.	
Effect measures	12	Specify for each outcome the effect measure(s) (e.g. risk ratio, mean difference) used in the synthesis or presentation of results.	Page 6
Synthesis methods	13a	Describe the processes used to decide which studies were eligible for each synthesis (e.g. tabulating the study intervention characteristics and comparing against the planned groups for each synthesis (item #5)).	Page 6
	13b	Describe any methods required to prepare the data for presentation or synthesis, such as handling of missing summary statistics, or data conversions.	Page 6
	13c	Describe any methods used to tabulate or visually display results of individual studies and syntheses.	Page 6
	13d	Describe any methods used to synthesize results and provide a rationale for the choice(s). If meta-analysis was performed, describe the model(s), method(s) to identify the presence and extent of statistical heterogeneity, and software package(s) used.	Page 6
	13e	Describe any methods used to explore possible causes of heterogeneity among study results (e.g. subgroup analysis, meta-regression).	Page 6
	13f	Describe any sensitivity analyses conducted to assess robustness of the synthesized results.	Page 6
Reporting bias assessment	14	Describe any methods used to assess risk of bias due to missing results in a synthesis (arising from reporting biases).	Page 6
Certainty assessment	15	Describe any methods used to assess certainty (or confidence) in the body of evidence for an outcome.	Page 6
**RESULTS**			
Study selection	16a	Describe the results of the search and selection process, from the number of records identified in the search to the number of studies included in the review, ideally using a flow diagram.	Page 8-9
	16b	Cite studies that might appear to meet the inclusion criteria, but which were excluded, and explain why they were excluded.	Page 8-9
Study characteristics	17	Cite each included study and present its characteristics.	Page 9-20
Risk of bias in studies	18	Present assessments of risk of bias for each included study.	Page 10-17

Section and Topic	Item #	Checklist item	Location where item is reported
Results of individual studies	19	For all outcomes, present, for each study: (a) summary statistics for each group (where appropriate) and (b) an effect estimate and its precision (e.g. confidence/credible interval), ideally using structured tables or plots.	Page 17-26
Results of syntheses	20a	For each synthesis, briefly summarise the characteristics and risk of bias among contributing studies.	Page 26-27
	20b	Present results of all statistical syntheses conducted. If meta-analysis was done, present for each the summary estimate and its precision (e.g. confidence/credible interval) and measures of statistical heterogeneity. If comparing groups, describe the direction of the effect.	Page 26-27
	20c	Present results of all investigations of possible causes of heterogeneity among study results.	Page 26-27
	20d	Present results of all sensitivity analyses conducted to assess the robustness of the synthesized results.	Page 26-27
Reporting biases	21	Present assessments of risk of bias due to missing results (arising from reporting biases) for each synthesis assessed.	Page 26-27
Certainty of evidence	22	Present assessments of certainty (or confidence) in the body of evidence for each outcome assessed.	Page 26-27
**DISCUSSION**			
Discussion	23a	Provide a general interpretation of the results in the context of other evidence.	Page 27-32
	23b	Discuss any limitations of the evidence included in the review.	Page 32-33
	23c	Discuss any limitations of the review processes used.	Page 32-33
	23d	Discuss implications of the results for practice, policy, and future research.	Page 33
**OTHER INFORMATION**			
Registration and protocol	24a	Provide registration information for the review, including register name and registration number, or state that the review was not registered.	Page 5<br><br>PROSPERO Registration n° CRD42024553896
	24b	Indicate where the review protocol can be accessed, or state that a protocol was not prepared.	Page 5
	24c	Describe and explain any amendments to information provided at registration or in the protocol.	N/A
Support	25	Describe sources of financial or non-financial support for the review, and the role of the funders or sponsors in the	Page 34

Section and Topic	Item #	Checklist item	Location where item is reported
		review.	
Competing interests	26	Declare any competing interests of review authors.	Page 24
Availability of data, code and other materials	27	Report which of the following are publicly available and where they can be found: template data collection forms; data extracted from included studies; data used for all analyses; analytic code; any other materials used in the review.	N/A

Table S2. Search strategies used for each database and number of records retrieved.

Database	Search Strategy	Number of Records Retrieved
PubMed (MEDLINE)	(("Lasers, Semiconductor"[MeSH] OR "Semiconductor Diode Lasers"[title] OR "Diode Laser, Semiconductor"[title] OR "Diode Lasers, Semiconductor"[title] OR "Laser, Semiconductor Diode"[title] OR "Lasers, Semiconductor Diode"[title] OR "Semiconductor Diode Laser"[title] OR "Diode Lasers"[title] OR "Diode Laser"[title] OR "Laser, Diode"[title] OR "Lasers, Diode"[title]) AND ("Vascular Malformations"[MeSH] OR "Malformations, Vascular"[title] OR "Malformation, Vascular"[title]) AND ("Mouth"[MeSH] OR "Oral Cavity"[title] OR "Cavity, Oral"[title] OR "Vestibule of the Mouth"[title] OR "Mouth Cavity Proper"[title]) AND ("Mouth Mucosa"[MeSH] OR "Mucosa, Mouth"[title] OR "Oral Mucosa"[title] OR "Mucosa, Oral"[title] OR "Buccal Mucosa"[title]) AND ("Pathology, Oral"[MeSH] OR "Oral and Maxillofacial Pathology"[title] OR "Oral Pathology"[title] OR "Pathology, Maxillofacial"[title] OR "Maxillofacial Pathology"[title]))	n = 103
Scopus	"Diode Laser" OR "Nd:YAG Laser" OR "CO2 Laser" AND "Vascular Malformations" OR "Oral Vascular Anomalies" AND "Oral Mucosa" OR "Gingiva" OR "Tongue"	n = 17
Web of Science (WoS)	"Lasers, Semiconductor" OR "Diode Lasers" OR "Diode Laser" AND "Vascular Malformations" OR "Oral	n = 19

	Vascular Lesions" AND "Oral Cavity" OR "Mouth Mucosa" OR "Buccal Mucosa"	

Table S3. Summary table of studies excluded in this systematic review [14–23].

**Excluded Studies**	**Exclusion Reasons**
Tempesta et al., 2023 [14]	Focused exclusively on pediatric population
Mungnirandr et al., 2016 [15]	Focused exclusively on pediatric population
Saliba et al., 2022 [16]	Case report with narrative review
Pauly et al., 2019 [18]	Focused exclusively on pediatric population
Marchand et al., 2022 [17]	Did not focused on the laser therapy
Couto et al., 2019 [19]	Did not focused on the laser therapy
Lim et al., 2023 [20]	Did not focused on the laser therapy
Sitra et al., 2014 [21]	Did not focused on the laser therapy
Junco et al., 2019 [22]	Did not focused on the oral mucosa
Goldenberg et al., 2017 [23]	Did not focused on the oral mucosa

Table S4. Criteria for judging risk of bias in ROBINS-I Version 2 (V2) tool [13].

Section/Domain	Description	Key Questions / Criteria	Response Options / Risk of Bias Judgement
A. Pre-assessment Screening	Determines whether further risk-of-bias assessment should proceed	A1. Was confounding addressed?<br>A2. If not, could unadjusted results be disregarded?<br>A3. Was outcome measurement inappropriate?	Y / PY / PN / N<br>→ Critical risk if A2 or A3 = Y or PY
B. Study Analysis Type	Identifies effect of interest	B1. Did follow-up differ by intervention received?<br>B2. Were intervention switches linked to outcome risk?	Y / PY / PN / N<br>Determines use of Domain 1 Variant A or B
C. Effect of Interest	Clarifies whether ITT or per-protocol effect is assessed	--	To assess: ITT (Variant A) or per-protocol (Variant B)
Domain 1: Bias due to Confounding	Assesses whether confounders were addressed	1.1 Controlled for confounders?<br>1.2 Confounders measured reliably?<br>1.3 Controlled for post-intervention vars?<br>1.4 Evidence of unmeasured confounding?	Y / PY / WN / SN / NI<br>Risk of bias: Low / Moderate / Serious / Critical
Domain 2: Bias in Classification of Interventions	Evaluates intervention misclassification risk	2.1 Post-follow-up classification?<br>2.2 Immortal time bias?<br>2.3 Pre-intervention data?<br>2.4 Influence of outcome knowledge?<br>2.5 Correct classification?	Y / PY / PN / N / NA / NI<br>Risk of bias: Low / Moderate / Serious / Critical
Domain 3: Bias in Selection of Participants	Selection process and timing issues	3.1–3.2 Immortal time bias?<br>3.3–3.4 Start of follow-up mismatch?<br>3.5–3.7 Post-intervention selection?<br>3.8–3.9 Adjustment/Sensitivity?<br>3.10 Sufficiently severe?	Y / PY / PN / N / NA / NI<br>Risk of bias: Low / Moderate / Serious / Critical

Domain 4 (VA): Bias due to Deviations from Intended Interventions (Assignment Effect)	Assesses treatment as assigned regardless of deviations	4.1 Experimental context?<br>4.2 Participant deviation due to recruitment?<br>4.3 Personnel undermining?<br>4.4 Deviations affect outcome?<br>4.5 ITT analysis appropriate?	Y / PY / PN / N / NA / NI<br>Risk of bias: Low / Moderate / Serious / Critical
Domain 4 (VB): Bias due to Deviations from Intended Interventions (Per-protocol Effect)	Assesses effect of adherence to assigned intervention	4.1 Adherence high?<br>4.2 Deviations affect outcome?<br>4.3 Per-protocol analysis appropriate?	Y / PY / PN / N / NA / NI<br>Risk of bias: Low / Moderate / Serious / Critical
Domain 5: Bias due to Missing Data	Impact and handling of missing data	5.1–5.3 Completeness of data?<br>5.4–5.5 Complete case analysis?<br>5.6–5.7 Missingness handled?<br>5.8–5.9 Imputation reasonableness?<br>5.10–5.11 Other methods or evidence?	Y / PY / PN / N / NA / NI / WN / SN<br>Risk of bias: Low / Moderate / Serious / Critical
Domain 6: Bias in Measurement of Outcome	Measurement consistency and assessor bias	6.1 Comparable measurement?<br>6.2 Assessor blinding?<br>6.3 Influence of knowledge of intervention?	Y / PY / PN / N / NA / NI / SY / WY<br>Risk of bias: Low / Moderate / Serious / Critical
Domain 7: Bias in Selection of Reported Result	Selective reporting and pre-specification	7.1 Pre-specified analysis?<br>7.2 Multiple outcome measures?<br>7.3 Multiple analyses?<br>7.4 Subgroup selection?	Y / PY / PN / N / NA / NI<br>Risk of bias: Low / Moderate / Serious / Critical
Overall Risk of Bias	Summary judgment from all domains	--	Low = all low<br>Moderate = ≥1 moderate

			only<br>Serious = ≥1 serious<br>Critical = ≥1 critical
Direction of Bias (if applicable)	Predicted bias direction if identifiable	Favors intervention / comparator<br>Upward / downward / towards / away from null	Optional, based on domain evidence

Table S5. Assessment of the risk of bias specific to each domain of the ROBINS-I V2 tool [1–4,13,24–29].

Reference First author et al. Year	D1 (VA)	D2	D3	D4 (VA)	D5	D6	D7
	1.1/1.2/1.3/1.4	2.1/2.2/2.3/2.4/2.5	3.1/3.2/3.3/3.4/3.5/3.6/3.7/3.8/3.9/3.10	4.1/4.2/4.3	5.1/5.2/5.3/5.4/5.5/5.6/5.7/5.8/5.9/5.10/5.11	6.1/6.2/6.3	7.1/7.2/73/7.4
Asai et al., 2014 [2]	WN WN N N	N NA Y N Y	N NA NI NA N NA NA NA NA NA	N NA NA NA NA	Y Y PY NA NA NA NA NA NA NA NA	N NI WY	PN N N N
Abukawa et al., 2017 [24]	PY PY N N	N NA Y N Y	N NA Y NA N NA NA NA NA NA	N NA NA NA NA	Y Y PY NA NA NA NA NA NA NA NA	N NI NA	PY PN PN N
Bacci et al., 2018 [4]	WN WN N N	N NA Y N Y	N NA Y NA N NA NA NA NA NA	N NA NA NA NA	Y Y PY NA NA NA NA NA NA NA NA	N NI WY	Y PN PY N
Miyazaki et al., 2018 [25]	WN WN N N	N NA Y N Y	N NA Y NA N NA NA NA NA NA	N NA NA NA NA	Y Y PY NA NA NA NA NA NA NA NA	N NI NA	Y PN PY N

Cadavid et al., 2018 [3]	WN	WN	N	N	N	NA	Y	N	Y	N	NA	Y	NA	N	NA	NA	NA	NA	NA	N	NA	NA	NA	NA	Y	Y	PY	NA	NA	NA	NA	NA	NA	NA	NA	N	NI	WY	PN	PN	PN	N
Limongelli et al., 2019 [26]	WN	WN	N	N	N	NA	Y	N	Y	N	NA	Y	NA	N	NA	NA	NA	NA	NA	N	NA	NA	NA	NA	Y	Y	PY	NA	NA	NA	NA	NA	NA	NA	NA	N	NI	WY	PY	PN	PN	N
Nammour et al., 2020 [5]	PY	PY	N	N	N	NA	Y	N	Y	N	NA	Y	NA	N	NA	NA	NA	NA	NA	N	NA	NA	NA	NA	Y	Y	PY	NA	NA	NA	NA	NA	NA	NA	NA	N	NI	NA	PN	N	N	N
Shivhare et al., 2022 [27]	PY	PY	N	N	N	NA	Y	N	Y	N	NA	NA	NA	N	NA	NA	NA	NA	NA	N	NA	NA	NA	NA	Y	Y	PY	NA	NA	NA	NA	NA	NA	NA	NA	N	NI	NI	Y	N	N	N
Bardhosh	WN	WN	N	N	N	NA	Y	N	Y	N	NA	NA	NA	N	NA	NA	NA	NA	NA	N	NA	NA	NA	NA	Y	Y	PY	NA	NA	NA	NA	NA	NA	NA	NA	N	NI	WY	PN	N	N	N

i et al., 2022 [28]							
Heimlich et al., 2023 [29]	WN WN NA N	N NA Y N Y	N NA NA NA N NA NA NA NA NA	N NA NA NA NA	Y Y PY NA NA NA NA NA NA NA NA	N NI WY	Y PN PN N
Gobbo et al., 2024 [1]	WN WN N N	N NA Y N Y	N NA NA NA N NA NA NA NA NA	N NA NA NA NA	Y Y PY NA NA NA NA NA NA NA NA	N NI WY	Y N N N

Abbreviations: D1: Domain 1 (Bias due to confounding), D2: Domain 2 (Bias in classification of interventions), D3: Domain 3 (Bias in selection of participants into the study or into the analysis), D4–VA: Variant A (Effect of assignment to intervention), D4: Domain 4 (Bias due to deviations from intended interventions), D5: Domain 5 (Bias due to missing data), D6: Domain 6 (Bias in measurement of the outcome), D7: Domain 7 (Bias in selection of the reported result)

Table S6. NHLBI Quality Assessment Tool for Case-Control Studies [2,3,5,12,25,26,28].

	NHLBI Quality Assessment Tool for Case-Control Studies												
**First Author et al., Year**	**Q1**	**Q2**	**Q3**	**Q4**	**Q5**	**Q6**	**Q7**	**Q8**	**Q9**	**Q10**	**Q11**	**Q12**	**Total Score**	**Quality Rating**
Asai et al., 2014 [2]	Y	Y	Y	NA	Y	NA	N	N	Y	Y	NR	Y	7/12 (58.33%)	Fair
Miyazaki et al., 2018 [25]	Y	Y	Y	NA	Y	NA	NR	N	Y	Y	NR	Y	7/12 (58.33%)	Fair
Cadavid et al., 2018 [3]	Y	Y	Y	NA	Y	NA	NR	N	Y	Y	NR	Y	7/12 (58.33%)	Fair
Limongelli et al., 2019 [26]	Y	Y	Y	NA	Y	NA	N	N	Y	Y	NR	Y	7/12 (58.33%)	Fair
Nammour et al., 2020 [5]	Y	Y	Y	NA	Y	NA	NR	N	Y	Y	NR	Y	7/12 (58.33%)	Fair
Bardhoshi et al., 2022 [28]	Y	Y	Y	Y	Y	Y	NR	Y	Y	Y	NR	Y	10/12 (83,33%)	Good

Q1: Was the research question or objective clearly stated and appropriate?, Q2: Was the study population clearly specified and defined?, Q3: Did the study include a justification for the sample size?, Q4: Were controls selected or recruited from the same or a comparable population as the cases (including the same time period)?, Q5: Were the definitions, inclusion and exclusion criteria, algorithms, or selection processes valid, reliable, and consistently

applied across all participants?, Q6: Were cases clearly defined and distinguishable from controls?, Q7: If less than 100% of eligible subjects were included, were cases and/or controls randomly selected from those eligible?, Q8: Were concurrent controls used?, Q9: Did investigators confirm that the exposure or risk factor occurred prior to the onset of the condition or event defining the case?, Q10: Were exposure/risk measures clearly defined, valid, reliable, and consistently applied across participants (including the same time period)?, Q11: Were assessors of exposure/risk blinded to the case/control status of participants?, Q12: Were key potential confounders measured and statistically adjusted for in the analysis? If matching was used, was it properly accounted for in the analysis?Total Score: Number of yes; CD: cannot be determined; NA: not applicable; NR: not reported; N: no; Y: yes. Quality Rating: Poor <50%, Fair 50–75%, Good ≥75%.

Table S7. NHLBI Quality Assessment Tool [12] for Observational Cohort and Cross-Sectional Studies [1,4,24,27,29].

	NHLBI Quality Assessment Tool for Observational Cohort and Cross-Sectional Studies														
**First Author et al., Year**	**Q1**	**Q2**	**Q3**	**Q4**	**Q5**	**Q6**	**Q7**	**Q8**	**Q9**	**Q10**	**Q11**	**Q12**	**Q13**	**Q14**	**Total Score**	**Quality Rating**
Abukawa et al., 2017 [24]	Y	Y	Y	Y	N	NA	Y	Y	Y	Y	Y	NR	Y	Y	11/14 (78.57%)	Good
Bacci et al., 2018 [4]	Y	Y	Y	Y	N	NA	Y	N	Y	Y	Y	NR	Y	Y	10/14 (71.42%)	Fair
Shivhare et al., 2022 [27]	Y	Y	Y	Y	N	NA	Y	Y	Y	Y	Y	NR	Y	Y	11/14 (78.57%)	Good
Heimlich et al., 2023 [29]	Y	Y	Y	Y	N	NA	Y	N	Y	N	Y	NR	Y	Y	9/14 (64.28%)	Fair
Gobbo et al., 2024 [1]	Y	Y	Y	Y	N	NA	Y	N	Y	N	Y	NR	Y	Y	9/14 (64.28%)	Fair

Q1: Was the research question or objective in this paper clearly stated?, Q2: Was the study population clearly specified and defined?, Q3: Was the participation rate of eligible persons at least 50%?, Q4: Were all subjects selected or recruited from the same or similar populations (including the same time period)? Were inclusion and exclusion criteria prespecified and applied uniformly to all participants?, Q5: Was a sample size justification, power description, or variance and effect estimates provided?, Q6: For the analyses in this paper, were the exposures of interest measured prior to the outcomes?, Q7: Was the timeframe sufficient to reasonably expect an association between exposure and outcome if one existed?, Q8: For exposures that vary in amount or level, did the study examine different levels of exposure in relation to the outcome (e.g., exposure categories or continuous measures)?, Q9: Were exposure measures (independent variables) clearly defined, valid, reliable, and consistently applied across all participants?, Q10: Was the exposure assessed more than once over time?, Q11: Were outcome measures (dependent variables) clearly defined, valid, reliable, and consistently applied across all participants?, Q12: Were outcome assessors blinded to the exposure status of participants?, Q13: Was loss to follow-up after baseline 20% or less?, Q14: Were key potential confounding variables measured and statistically adjusted for in assessing the relationship between exposures and outcomes?Total Score: Number of yes; CD: cannot be determined; NA: not applicable; NR: not reported; N: no; Y: yes. Quality Rating: Poor <50%, Fair 50–75%, Good ≥75%.
